# Anterior inferior cerebellar artery (AICA) aneurysms: a radiological study of 15 consecutive patients

**DOI:** 10.3389/fradi.2023.1229921

**Published:** 2023-08-08

**Authors:** Sajjad Muhammad, Ahmad Hafez, Hanna Kaukovalta, Behnam Rezai Jahromi, Riku Kivisaari, Daniel Hänggi, Mika Niemelä

**Affiliations:** ^1^ Department of Neurosurgery, University of Helsinki and Helsinki University Hospital, Helsinki, Finland; ^2^ Department of Neurosurgery, Medical Faculty and University Hospital Düsseldorf, Heinrich-Heine-University Düsseldorf, Düsseldorf, Germany; ^3^ Department of Neurosurgery, King Edward Medical University, Lahore, Pakistan

**Keywords:** AICA aneurysms, characteristics of AICA aneurysms, CT angiography, radiological features, clinical outcome

## Abstract

**Introduction:**

The aneurysms of the anterior inferior cerebellar artery (AICA) are rare lesions of the posterior circulation and to treat them is challenging. We aim to present anatomical and morphological characteristics of AICA aneurysms in a series of 15 patients.

**Method:**

The DSA and CT angiography images of AICA aneurysms in 15 consecutive patients were analyzed retrospectively. Different anatomical characteristics were quantified, including morphology, location, width, neck width, length, bottleneck factor, and aspect ratio.

**Results:**

Eighty percent of the patients were females. The age was 52.4 ± 9.6 (mean ± SD) years. 11 patients were smokers. Ten patients had a saccular aneurysm and five patients had a fusiform aneurysm. Aneurysm in 10 patients were located in the proximal segment, in three patients in the meatal segment, and in two patients in the distal segment. Ten out of 15 patients presented with a ruptured aneurysm. The size of AICA aneurysms was 14.8 ± 18.9 mm (mean ± SD). The aspect ratio was 0.92 ± 0.47 (mean ± SD) and bottleneck factor was 1.66 ± 1.65 (mean ± SD).

**Conclusion:**

AICA aneurysms are rare lesions of posterior circulation predominantly found in females, present predominantly with subarachnoid hemorrhage, and are mostly large in size.

## 1. Introduction

The aneurysm of the anterior inferior cerebellar artery (AICA) are very rare and they account for <1% of all intracranial aneurysms (1, 2). The reported case reports of AICA aneurysms have a single or a few patients (1, 3–62), except for a few larger case series (63), including a large series of AICA aneurysms reported by Drake and colleagues (64). However, in this series, the aneurysms originating from the basilar trunk that is near the origin of AICA were included. AICA aneurysms are more often large in size, which makes their surgical and endovascular treatments challenging. Coiling is the favored treatment of posterior circulation aneurysms (65). However, in selected cases surgery might be a better choice, particularly in larger sized AICA aneurysms (26, 57). The surgical approach depends on location of aneurysm in relation to clivus. Aneurysms laying high can be approached through sub-temporal approach and low laying aneurysms are approached through retro-sigmoidal corridor. The morphological features may be helpful in deciding upon a treatment strategy. Due to the rarity of these lesions, the anatomical and radiological characteristics in unselected patient cohorts with AICA aneurysms have seldom been reported. In this series, we report the radiological features including shape, size, aspect ratio (maximum perpendicular height divided by neck diameter), bottleneck factor (maximum height to width ratio), and frequencies of aneurysm location in different segments of AICA aneurysms in a medium-size series. We report 15 consecutive patients harboring AICA aneurysms admitted from 1968 to 2017 in Helsinki University Hospital, department of Neurosurgery.

## 2. Methods

### 2.1. Patients and radiological data

Through retrospective analysis of our aneurysm data base, a total of 21 patients harbouring AICA aneurysms were identified. Six patients were excluded because of missing CT angiography scans or DSA. The remaining 15 patients harboring AICA aneurysms were included in the study. A 4-slice scanner (GE Lightspeed QX/I; GE Medical Systems, Milwaukee, Wisconsin, USA) was used for analyzing CT angiography images until 2007. Since then, a 32-slice scanner (GE LightSpeed Pro 32) or a 64-row scanner (GE lightSpeed VCT Advantage) was used to analyze CT angiography images. The digital archiving system of the hospital (IMPAX, version 5.3, Agfa, Mortsel, Belgium) was used to store the images. Two board-certified neurosurgeons analyzed the CTA scans.

The study was approved by the local ethical committee. The ethical number for the aneurysm database is HUS/197/2016. For retrospective data collection, an additional approval/consent was not required.

### 2.2. Radiological measurements

For each aneurysm, the maximal length, width, neck diameter, shape (saccular/fusiform), and the location at different segments of AICA were quantified. The above measurements were used to calculate aspect ratio and bottleneck factor.

### 2.3. Statistical analysis

The commercial statistical software IBM SPSS (version 20.0.0.) was used to analyze the data. The data is presented as mean ± SD or in frequencies (percentage).

## 3. Results

### 3.1. Patients and aneurysms

In our series (from 1968 to 2017), a total of 9,832 patients harboring intracranial aneurysms were studied. 21 patients out of 9,832 had AICA aneurysms showing an incidence of AICA aneurysms occurrence in about 0.22% of all intracranial aneurysms. Due to missing proper scans, we could analyze AICA aneurysms in 15 patients. Out of the 15 patients with AICA aneurysms, 12 (80%) were female and 3 (20%) were males. The mean age of the patients was 52.4 ± 9.6 years. In this case series, 11 (73%) of the patients were smokers. Also, 11 (73%) of the patients had a ruptured aneurysm (Table 1).

**Table 1:** Radiological characteristics of anterior inferior cerebellar artery aneurysms.

	Total number	Percentage
Number of patients	15	
Age, years (mean ± SD)	52.4 ± 9.6	
Males	3	20
Females	12	80
Current smoker	11	73.3
Non-smoker	4	26.7
Presentation with SAH	11	73.3
Non-SAH	4	26.7
Cranial nerve deficits		
Yes	5	33.3
No	10	66.7
Shape		
Saccular	10	66.7
Fusiform	5	33.3
Side		
Left	7	46.7
Right	8	53.3
Location		
Proximal	10	66.6
Meatal	3	20
Distal	2	13.3

### 3.2. Radiological characters of AICA aneurysms

Ten (67%) patients had a saccular aneurysm and five (33%) patients had a fusiform aneurysm. Ten (67%) aneurysms were located in the pre-meatal segment, three (20%) aneurysms were located in the meatal segment, and the two (13%) aneurysms were located in the post-meatal segment. The AICA aneurysms had a mean size of 14.8 ± 18.9 mm. The ACIA aneurysm had a mean aspect ratio of 0.92 ± 0.47 and they had a mean bottleneck factor of 1.66 ± 1.65 (Table 2). Interestingly, in the proximal segment 30% and in the distal segments 40% of the aneurysms were fusiform (Tables 1, 2).

**Table 2:** Radiological characteristics of anterior inferior cerebellar artery aneurysms (*n* = 15).

Radiological characteristics	Mean ± SD
Aneurysm size (mm), (*n* = 15)	14.8 ± 18.9
Width (mm)	14.8 ± 18.9
Length (mm)	16.9 ± 20.3
Neck (mm)	12.6 ± 13.1
Aspect ratio of saccular aneurysms (*n* = 10)	0.92 ± 0.47
Bottleneck factor of saccular aneurysms (*n* = 10)	1.66 ± 1.65

### 3.3. Clinical presentation and outcomes

Out of 15 patients, 11 (63.3%) presented with subarachnoid hemorrhage, and 4 (26.7%) patients with incidental findings. Among the ruptured cases 9 out of 11 (81.8%) presented with a good grade SAH (Hunt and Hess grade 1–3) and the remaining 2 out of 11 patients presented with a poor grade SAH (Hunt and Hess 4–5). In radiological analysis 3 out of 15 patients presented with intracerebral hematoma and 6 out of 15 patients presented with intraventricular hemorrhage as reported by Muhammad et al. in 2023. 11 out of 15 patients were treated surgically, only one patient was treated with endovascular coiling and 3 patients remain conservative. One of the conservatively treated patient lived more than 40 years and finally died in 2012 due to other systemic diseases and not due to SAH. In other two cases, patients were admitted with poor grade SAH and did not survive from severe SAH. All the unruptured aneurysms had good clinical outcomes (mRS 1–2) on discharge. In patients with ruptured AICA aneurysms, 9 out of 11 patients (81.8%) had good clinical outcomes (mRS 1–2) and 2 out of 11 patients (18.2%) had poor clinical (mRS 3–6) outcomes (66).

## 4. Discussion

**Figure 1:**
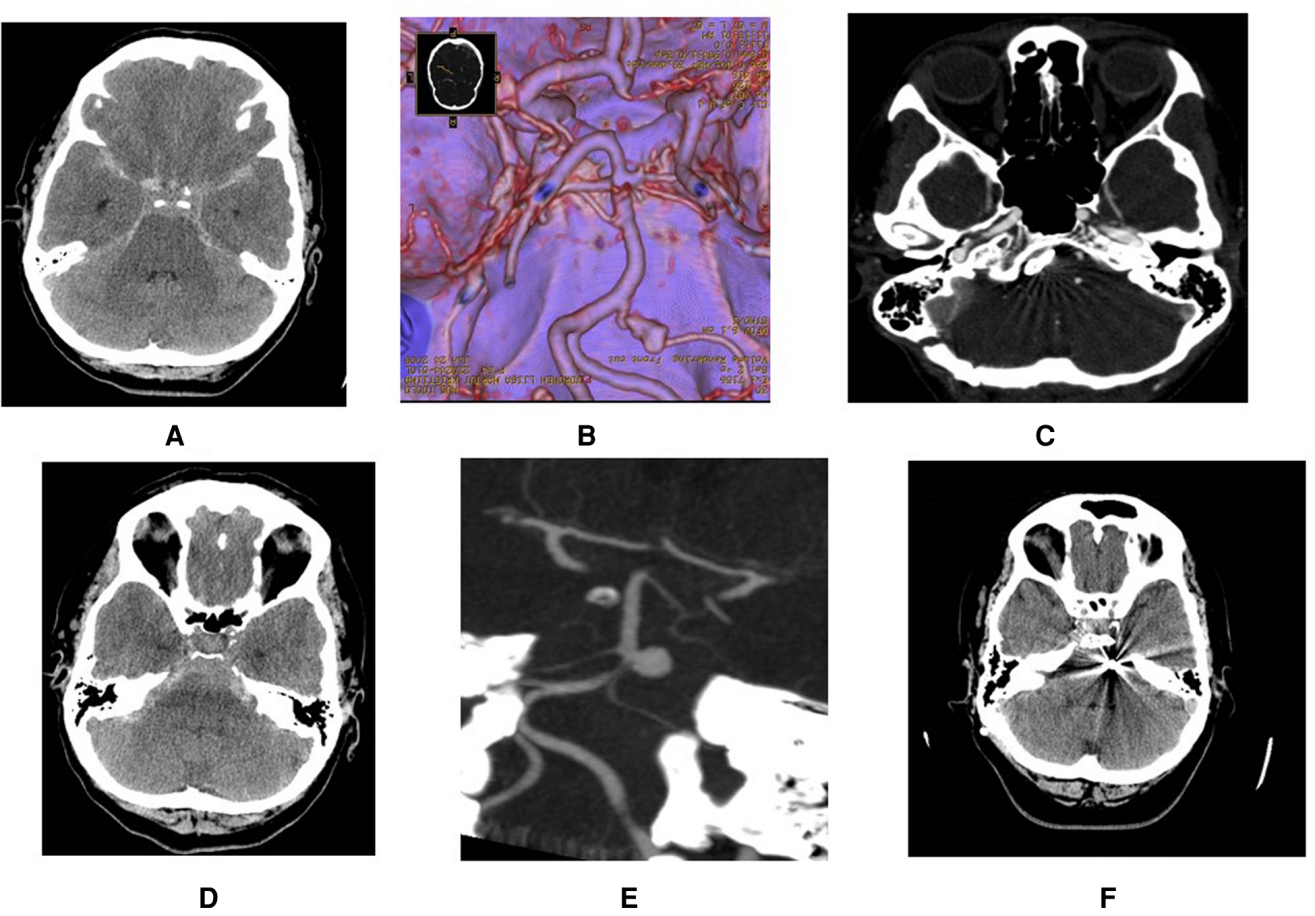
Demonstration of a ruptured AICA aneurysms at proximal location (**A,B**) that was treated surgically through retrosigmoid approach (**C**). Another patients with ruptured high laying AICA aneurysm (**D,E**) that was treated through sub-temporal approach (**F**).

**Figure 2:**
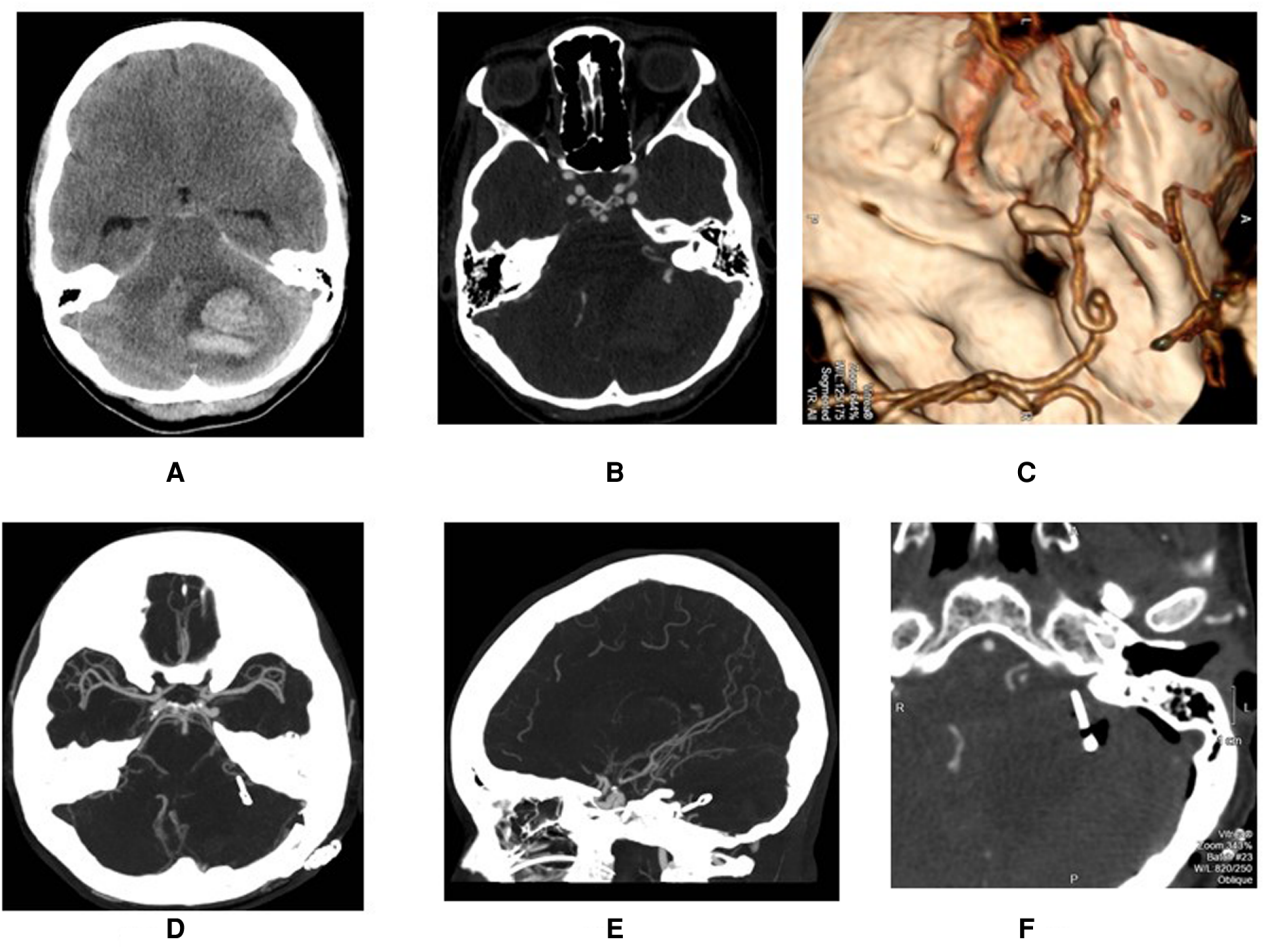
Demonstration of a ruptured AICA aneurysms with intra cerebral hemorrhage (**A–C**) that was treated through retrosigmoid approach (**D–F**).

**Figure 3:**
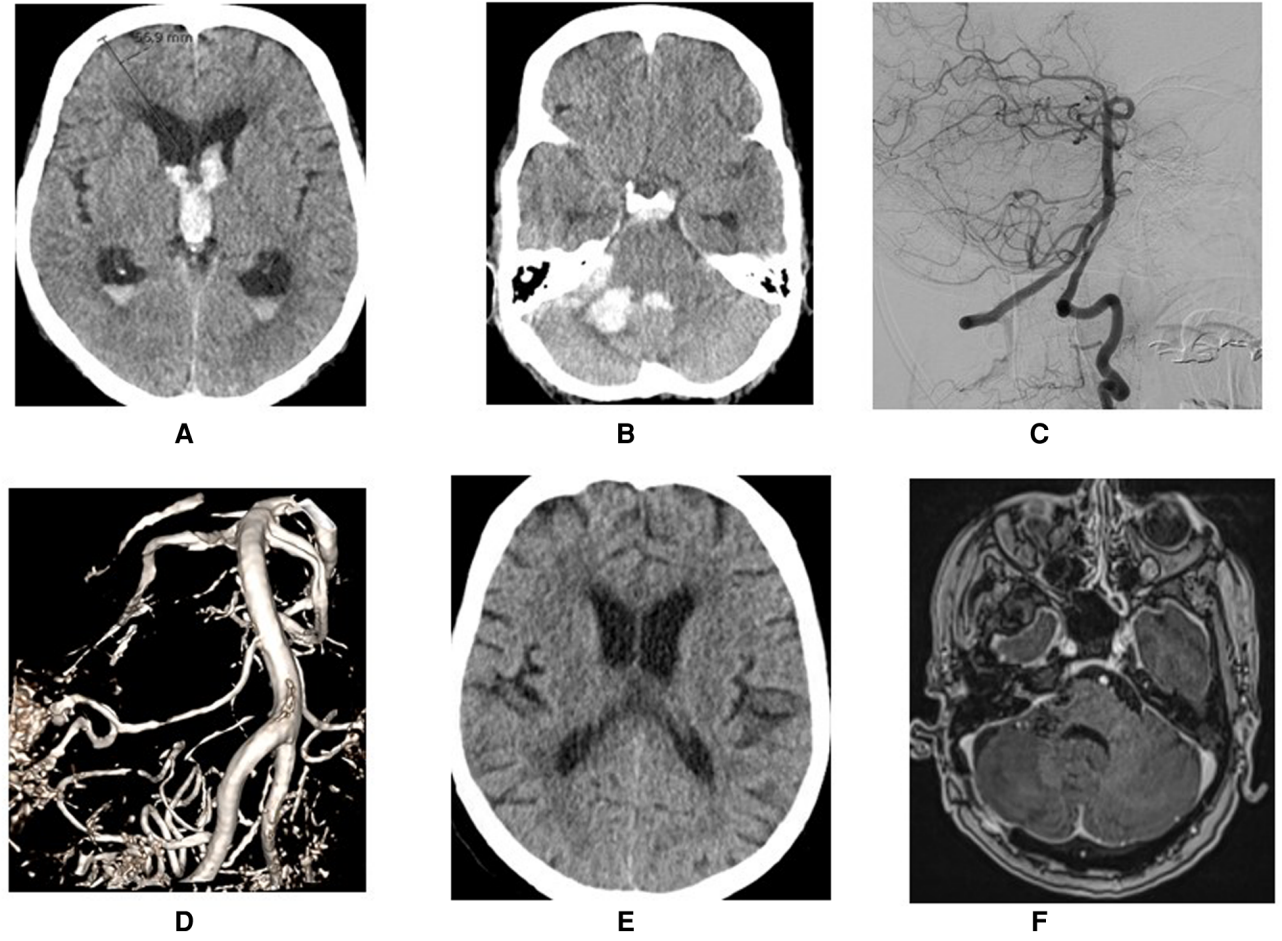
Demonstration of a ruptured AICA aneurysms at meatal loop with intra cerebral hemorrhage (**A–C**) that was treated through retrosigmoid approach (**D–F**).

AICA aneurysms are rare and have been reported in <1% of all intracranial aneurysms (1, 2). Our data show that the incidence of AICA aneurysms in an unselected cohort was 0.22%. The most common presentation of an AICA aneurysm is subarachnoid hemorrhage (SAH). Seventy three percent of patients in our series presented with SAH, which is similar to previously published data (63). A review of data from already published case reports of 56 patients revealed that 81% of patients presented with SAH, which is slightly greater than our series and the series presented previously. Patients with more insidious onset present with cranial nerve deficits very similar to cerebellopontine angle space occupying lesions. However, patients with SAH may also have cranial nerve deficits often in the seventh and eighth cranial nerves. In our series, 33% (*N* = 5) of the patients had cranial nerve (VI, VII, VIII, or IX) deficits (Table 1). Rarely, patients with an AICA aneurysm present with convulsive seizure (36). In our series, none of the patients presented with epilepsy. AICA can be anatomically divided into four segments including (67) anterior pontine (a1), lateral pontine (a2), flocculopeduncular (a3), and cortical (a4). The lateral pontine segment has a close relationship with the porus of the internal acoustic meatus and with the facial and cochleovestibular nerves. AICA aneurysms can be categorized into proximal, meatal, and distal (58). Our data shows that 67% (*n* = 10) of the aneurysms were located proximally, 20% (*n* = 3) were in the meatal region, and only 13% (*n* = 2) were located distally. Our data are consistent with a previously published short case series where five out of six aneurysms were located proximally (1). Meatal aneurysms can be further classified into three subtypes (58). Type I (also called remote) are localized in the vascular loop outside the meatus. Type II (plugged) aneurysms are partially buried in the internal-acoustic meatus, and type III (buried) are entirely contained by the internal auditory canal). In our series, we found three aneurysms that were located in the meatal segment. Two of these were type I and one was type II (not shown in the table). The location of the aneurysm facilitates the planning of a suitable surgical approach if surgery is indicated (Figures 1–3). Most of the AICA aneurysms are reached through retrosigmoid approach (Figures 1–3). The mean ± SD size of the AICA aneurysms was 14.8 ± 18.9. The mean ± SD width was 16.9 ± 20.3 mm and the mean ± SD length was 16.9 ± 20.3 (Table 2). It is noteworthy that 73% of the patients were smokers and smoking is a potential risk factor for aneurysm formation and rupture. Further analysis of saccular aneurysms (*n* = 10) revealed a mean ± SD aspect ratio of 0.92 ± 0.47 and a mean ± SD bottleneck factor of 1.66 ± 1.65 (Table 2). Our data demonstrate that most AICA aneurysms are larger in size, which is consistent with a previous report that analyzed 10 thrombosed AICA aneurysms (20). In most cases in this series, the aneurysm size was >10 mm; two of these aneurysms were giant.

## 5. Conclusion

AICA aneurysms are rare posterior circulation lesions predominantly found in females, present predominantly with subarachnoid hemorrhage, are mostly large in size.

## 6. Limitations

Our analysis was retrospective and may not completely reflect the natural history of anatomical and radiological parameters. The aspect ratio was quantified in a few cases after rupture and hence the possibility remains that the aspect ratio might have changed due to morphological changes after rupture and to a compression effect due to subarachnoid blood. Due to the small number of patients, we could not analyze the influence of aspect ratio, bottleneck factor, and segmental location of the aneurysm on aneurysm rupture.

## Data Availability

The original contributions presented in the study are included in the article, further inquiries can be directed to the corresponding author.
